# Metastasis of Breast Tumor Cells to Brain Is Suppressed by Phenethyl Isothiocyanate in a Novel *In Vivo* Metastasis Model

**DOI:** 10.1371/journal.pone.0067278

**Published:** 2013-06-27

**Authors:** Parul Gupta, Chris Adkins, Paul Lockman, Sanjay K. Srivastava

**Affiliations:** 1 Department of Biomedical Sciences, Texas Tech University Health Sciences Center, Amarillo, Texas, United States of America; 2 Cancer Biology Center, Texas Tech University Health Sciences Center, Amarillo, Texas, United States of America; 3 Department of Pharmaceutical Sciences, Texas Tech University Health Sciences Center, Amarillo, Texas, United States of America; Enzo Life Sciences, Inc., United States of America

## Abstract

Breast tumor metastasis is a leading cause of cancer-related deaths worldwide. Breast tumor cells frequently metastasize to brain and initiate severe therapeutic complications. The chances of brain metastasis are further elevated in patients with HER2 overexpression. In the current study, we evaluated the anti-metastatic effects of phenethyl isothiocyanate (PEITC) in a novel murine model of breast tumor metastasis. The MDA-MB-231-BR (BR-brain seeking) breast tumor cells stably transfected with luciferase were injected into the left ventricle of mouse heart and the migration of cells to brain was monitored using a non-invasive IVIS bio-luminescent imaging system. In order to study the efficacy of PEITC in preventing the number of tumor cells migrating to brain, mice were given 10 µmol PEITC by oral gavage for ten days prior to intra-cardiac injection of tumor cells labeled with quantum dots. To evaluate the tumor growth suppressive effects, 10 µmol PEITC was given to mice every day starting 14^th^ day after intra-cardiac cell injection. Based on the presence of quantum dots in the brain section of control and treated mice, our results reveal that PEITC significantly prevented the metastasis of breast cancer cells to brain. Our results demonstrate that the growth of metastatic brain tumors in PEITC treated mice was about 50% less than that of control. According to Kaplan Meir’s curve, median survival of tumor bearing mice treated with PEITC was prolonged by 20.5%. Furthermore as compared to controls, we observed reduced HER2, EGFR and VEGF expression in the brain sections of PEITC treated mice. To the best of our knowledge, our study for the first time demonstrates the anti-metastatic effects of PEITC *in vivo* in a novel breast tumor metastasis model and provides the rationale for further clinical investigation.

## Background

Tumor metastasis is a significant clinical problem, which aggravates the complexity of cancer pertaining to therapeutic purposes and responsible for about 90% of all cancer deaths [1], [2]. The clinical efficacy of therapeutic modalities is further hampered by the presence of metastatic tumors. In women about 23% of all diagnosed cases of cancer are of breast cancer, which is the highest amongst all the cancers accounting for about 7.6 million deaths worldwide each year [3]. One of the major causes of reduced survival in breast cancer patients is early tumor metastasis to different organs [2], [4], [5].

Although, the treatment strategies for breast cancer are evolving with time but a recent study shows that incidence of brain metastasis is still rising in the patients [6]. It has been reported that, 10–16% of breast cancer patients present the problem of brain metastasis [7]. The efficacy of treatment therapies is reduced in patients having metastasized tumors to brain. Furthermore, tumor metastasized to brain also present neurologic complications which may not be completely reversed with treatment [7]–[9]. Accumulating evidences suggest a correlation between high expression of HER2 and VEGF with increased brain metastasis of breast cancer cells leading to reduced survival rates [10]–[16]. The problems associated with the tumor metastasis emphasize the need of therapies that can help in prevention of metastasis development and growth and improving the quality of life.

There are several epidemiological reports from different parts of the world indicating an inverse correlation between intake of cruciferous vegetables and occurrence of breast cancer [17]. A recent epidemiological study also suggested that the intake of cruciferous vegetables was associated with reduced risk of breast cancer in African American women [18]–[20].

Phenethylisothiocyanate (PEITC) is an active ingredient of cruciferous vegetables and has shown anti-cancer effects against several cancers [21]–[31]. Interestingly, this compound is in the clinical trials for lung cancer and lymphomas. In our previous studies, we demonstrated the anti-cancer effects of PEITC against breast cancer via HER2 suppression [32]. However, the anti-metastatic potential of this phytochemical has not been fully explored yet and is the focus of the present study.

## Materials and Methods

### Ethics Statement

The use of animals and treatment was approved by Institutional Animal Care and Use Committee (IACUC) of Texas Tech University Health Science Center. Experiments were conducted in strict compliance with IACUC regulations. The mice showing signs of distress, pain and suffering due to tumor burden were humanely sacrificed.

### Brain Metastasis Model

Female athymic nude mice (4–6 weeks old) were obtained from Charles River (Wilmington, MA, USA) and maintained under specific pathogen-free conditions. The use of athymic nude mice and their treatment was approved by the Institutional Animal Care and Use Committee (IACUC), Texas Tech University Health Sciences Center, and the experiments were conducted in strict compliance with the regulations. Mice were given antioxidant-free AIN-76A special diet (Test Diet, Richmond, IN, USA) one week before starting the experiment as described by us previously [33], [34]. MDA-MB-231 (BR) (BR: brain seeking) breast cancer cells transfected with luciferase plasmid were selected for brain specificity by successive cycles of *in vivo* selection and *in vitro* culture [35]. Kind gift of MDA-MB-231 (BR) cells and HER2 overexpressing MDA-MB-231 (HH) cells by Dr. Patricia S. Steeg (National Cancer Institute, Maryland) and Dr. Quentin Smith (Texas Tech University Health Sciences Centre, Amarillo, Texas) are greatly appreciated. These cell lines were used by us and others previously [10], [32], [36], [37]. We injected these cells in mice as per method originally described by Conley et al. [38] and later by us [36], [37]. Briefly MDA-MB-231 (BR) cells expressing luciferase were harvested, washed and re-suspended in sterile phosphate buffered saline (PBS) at a density of 0.2×10^6^ cells/100 µl. These cells were injected into the left ventricle of the heart of each mouse under anesthesia. Each mouse was imaged using IVIS Lumina imaging system (Calipers, Hopkinton, MA, USA) after intra-peritoneal injection of 3 mg luciferin (Goldbio., St. Loius, MO, USA) in 100 µl of sterile water. The luminescence from cells injected was observed in brain within 5 minutes of intracardiac injection of cells but the signal decayed gradually by 5–10 days. However, signal starts reappearing two weeks after the tumor cell injection and was retained by 70% of the mice by day 40, suggesting that MDA-MB-231 (BR) cells were growing *in vivo*.

### Metastasis Prevention Model

In this experiment, mice were orally gavaged with 10 µmol PEITC in 100 µl PBS every day as described by us earlier [39]. After 10 days of PEITC treatment, intra-cardiac injection of MDA-MB-231 (BR) cells was given to these mice as described above. Prior to injection cells were labeled with Qtracker 800 (Invitrogen, Grand Island, NY, USA) as per the manufacturer’s instructions. The PEITC treatment continued for another 10 days after cell injection, while control animals were given vehicle alone for same time period. Both control and treated group had 6 mice each. The mice were imaged periodically for the signal in brain using non-invasive IVIS Lumina system. At the end of the experiment mice were euthanized and the brain was removed carefully and fixed in 4% paraformaldehyde overnight at room temperature. Care was taken not to damage the brain tissue. Next day brains were transferred into 30% sucrose solution and stored at 4°C overnight. The brains were then removed from sucrose solution and frozen at −20°C. Sections of 20 µm thickness were made from the frozen brains using cryostat (Leica, Buffalo Grove, IL, USA). Quantum dots in each brain were counted under fluorescence microscope (Olympus Inc., Center Valley, PA, USA) using excitation wavelength of 405–760 nm. At least 50 sections from each brain were counted for quantum dots. The brain sections of at least three mice from each group were analyzed.

### Metastasized Tumor Growth Suppression Model

In this experiment, MDA-MB-231 (BR) cells were injected into the heart’s left ventricle of each mouse as described above and each mouse was imaged periodically. Two weeks after the tumor cell injection, mice were randomly divided into two groups with 10 mice per group. In the treated group, each mouse was given 10 µmol PEITC and control group was administered with vehicle alone by oral gavage everyday till the duration of the experiment. The control group received vehicle only. Only those mice which showed luminescence in the brain area during the duration of the experiment were included in the overall analysis. The mice were sacrificed at day 39 and their brains were removed carefully. The brains were processed for immunostaining for HER2, EGFR and VEGF.

### Immunostaining of Brain Sections

The brains were snap frozen in isopentane at −80°C and 2 µm sections were prepared from the brains using a cryostat. The sections were gently placed on positively charged slides, fixed using 4% paraformaldehyde and permeabilized with 0.2% Triton X-100. The sections were then blocked with 5% goat serum for 60 minutes. After blocking, sections were probed with primary antibodies specific to HER2 (#ab2428, Abcam, Cambridge, MA, USA; 1∶100), EGFR (# 2232, Cell Signaling Technology, Danvers, MA, USA; 1∶200) and VEGF (#MAB3045, R&D Systems, Minneapolis, MN; 1∶500) overnight at 4°C, followed by incubation with Alexa Flour 488 (Anti-mouse) (A11001, Life Technologies, Grand Island, NY, USA) for VEGF and Alexa Flour 594 (Anti-rabbit) (A11037, Life Technologies, Grand Island, NY, USA) for EGFR and HER2 for 1 h with gentle rocking at room temperature. After washing the sections were counterstained with DAPI for nuclear staining as internal control. Brain sections were photographed under fluorescence microscope (Olympus Inc., Center Valley, PA, USA) after coverslips were mounted on each slide. The expression of HER2, EGFR and VEGF was quantitated using SlideBook software (Intelligent Imaging Innovations, Inc., Denver, CO, USA).

### Survival Model

In this study, MDA-MB-231 (BR) cells were injected into the heart’s left ventricle of each mouse as described above and each mouse was imaged periodically. Two weeks after the tumor cell injection, mice were randomly divided into two groups with 10 mice per group. In the treated group, each mouse was given 10 µmol PEITC by oral gavage everyday till the duration of the experiment. These mice were monitored regularly for survival until all the mice in control group were dead. The time and number of deaths in both the groups were recorded regularly. The experiment was conducted under the strict compliance of IACUC. The mice showing signs of distress, pain and suffering due to tumor burden were humanely sacrificed. Data was plotted on Kaplan Meir’s survival curve using Prism 5.0 software (GraphPad software Inc., San Diego, CA, USA). This curve was used to analyze the survival pattern of mice in control and treatment groups.

### Cell Culture

Human breast carcinoma cell lines MDA-MB-231 (BR) Luc2 and the MDA-MB-231 (BR) cells with HER2 overexpression were kindly provided by Dr. Patricia Steeg (NIH, Bethesda, MD, USA) and Dr. Quentin Smith (Texas Tech University Health Sciences Center, Amarillo, TX, USA). These cells were maintained in DMEM supplemented with 10% FBS and 5% PSN [10]. The HER2 overexpressing cells MDA-MB-231 (HH) were maintained in the medium described above in the presence of 300 µg/ml zeocin. All the cells used in this study were within twenty passages after receipt or resuscitation. The cells were maintained and passaged in culture as described by us previously [39].

### Transwell Cell Invasion Assay

Cell invasion was performed according to manufacturer’s instructions in Boyden’s Transwell chamber with 8.0 µm pore size filters (BD Biosciences, San Jose, California, USA) and as described by us earlier [40]. Briefly, cells were serum starved overnight and collected after trypsinization. About 20×10^3^ cells (300 µl) containing 1% serum was seeded on the upper well of Boyden’s chamber and the lower chamber was filled with 1.0 mL DMEM medium containing 1% serum. After incubation for 2 h, 5 µM of PEITC was added to upper compartment of the Boyden’s chamber while the medium in lower chamber was replaced with DMEM containing 10% FBS and 20 ng/ml of VEGF as chemo-attractant. After incubation for 24 hours, cells from the upper chamber were removed by wiping with a cotton swab. The stained membranes were removed from the transwell and transferred into the individual wells of a 96-well plate and stained using 0.4% sulforhodamine B (SRB) solution in 1% acetic acid. The cells were fixed with 10% tricholoroacetic acid at 4°C for 1 hour and washed with 1% acetic acid solution. The SRB dye retained on the membrane was solubilized with 10 mM Tris buffer and the absorbance was read at 570 nm using a microplate reader (BioTek Instruments, Winooski, VT, USA). Assays were performed in triplicates and data was expressed as percent migration compared with control.

### Statistical Analysis

Statistical analysis was performed using Prism 5.0 (GraphPad software Inc., San Diego, CA, USA). Results were represented as means ± SD or S.E.M. Data was analyzed by Student’s *t*-test. Differences were considered statistically significant at p<0.05.

## Results

### PEITC Reduces Brain Metastasis of Breast Cancer

**Figure 1:**
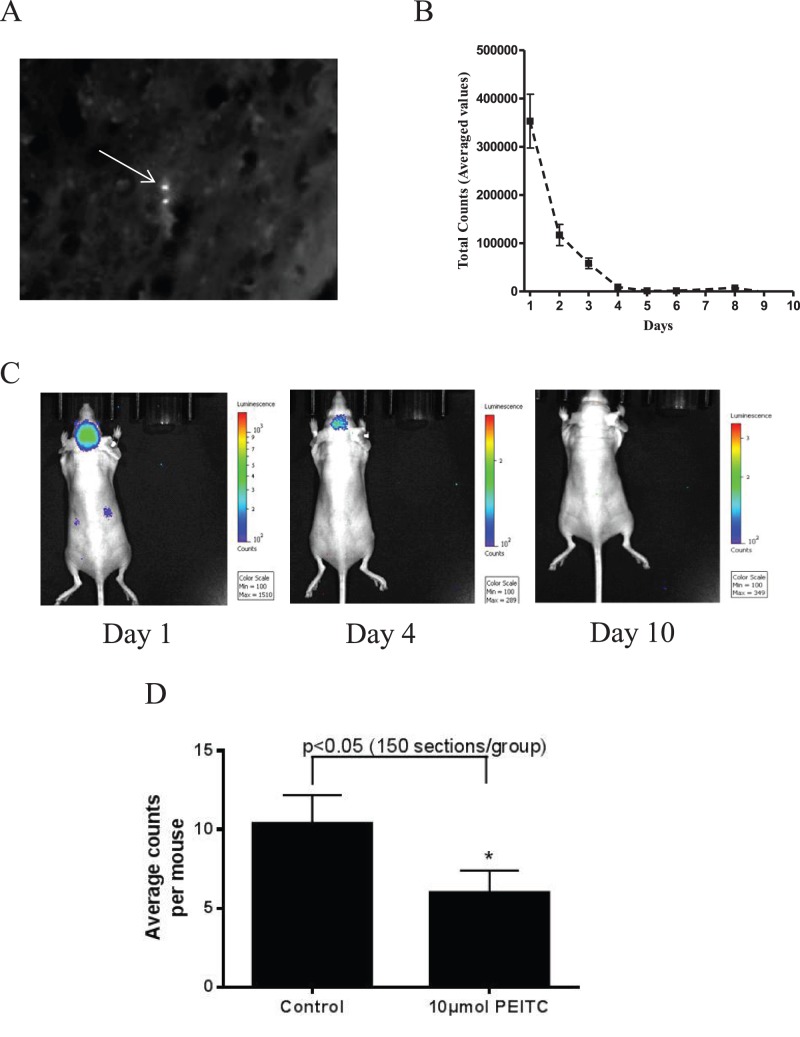
Reduction of brain metastasis. (A) Presence of MDA-MB-231 (BR) breast cancer cell labeled with quantum dot in the brain of mice as seen under fluorescent microscope. (B) Luminescence decay curve from mice brain starting the day of cell injection till day 10 after intra-cardiac injection of MDA-MB-231 (BR) breast cancer cells. (C) Mice brain images as analyzed by IVIS Lumina imaging system at different time points after intra-cardiac injection of MDA-MB-231 (BR) cells. (D) Average count of quantum dot labeled MDA-MB-231 (BR) tumor cells in mice brain of control and PEITC (10 µmol) treated groups. Values are represented as means±SEM. * P<0.05, statistically different when compared with control. At least 150 brain sections from each group were analyzed.

In most of the breast cancer patient’s brain is the major site for metastasis. We first wanted to see if PEITC can suppress the migration of breast cancer cells to brain. To address this question, MDA-MB-231 (BR) cells were tagged with quantum dots and then these cells were injected into the left ventricle of the heart of athymic nude mice, which were pretreated with 10 µmol PEITC by oral gavage for 10 days. Kinetics of the injected cells was monitored by non-invasive IVIS bio-imaging system. Tumor cells were lodged into the brain within 5–10 min of intra-cardiac injection, as indicated by luminescence. However, the signal in brain decreased gradually and eventually vanished by 5–10 days (Fig. 1B&C). At day 10, mice were euthanized and brains were collected from control and treated groups. The 20 µm sections of the brains were analyzed by counting quantum dots in a blinded manner (Fig. 1A). Based on the average counts of quantum dots in each mouse brain, our results showed about 50% reduction in brain metastasis of breast cancer cells in PEITC treated group, as compared to controls (Fig. 1D). These results suggest the potential of PEITC in blocking the metastasis of breast cancer cells to brain by inhibiting the seeding capability of metastatic cells.

### PEITC Suppresses the Growth of Metastasized Breast Tumors

**Figure 2:**
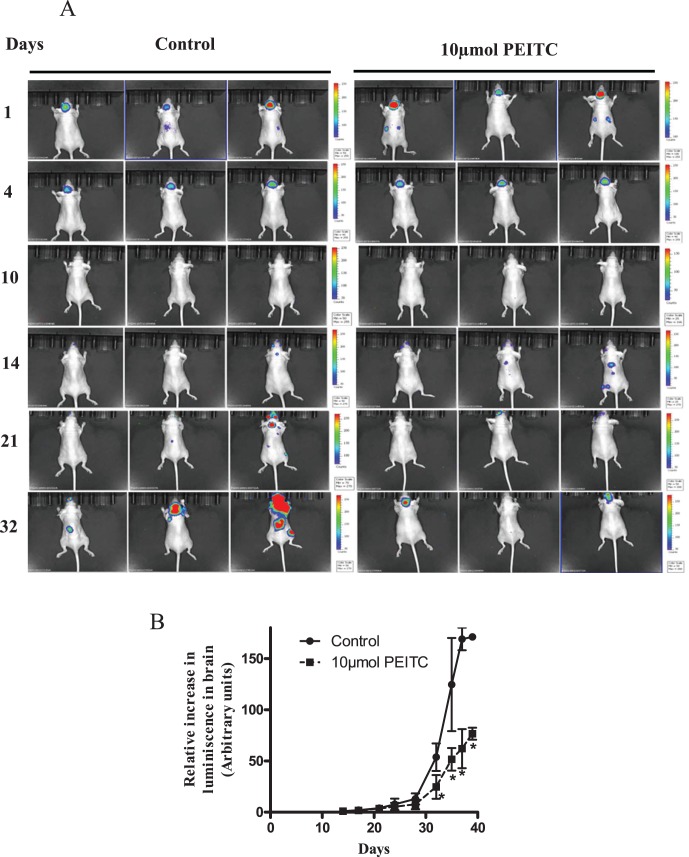
Growth suppression of tumor cells in brain. (A) The MDA-MB-231 (BR) breast cancer cells that reach brain start developing tumors after 14^th^ day of intra-cardiac injection. The PEITC (10 µmol by oral gavage) treatment started on 14^th^ day of tumor cell implantation and mice were imaged periodically. Luminescence signal from brain was collected using IVIS *in vivo* animal imager. (B) Average luminescence after quantification of the signal from mice brain and plotted against time (days) to obtain tumor growth curve. The arbitrary units were used for luminescence intensity quantification. The change in signal intensity from each mouse brain was calculated relative to the initial signal observed on day 14. * P<0.05, statistically different when compared with control. Results are presented as mean ± SD of triplicates.

**Figure 3:**
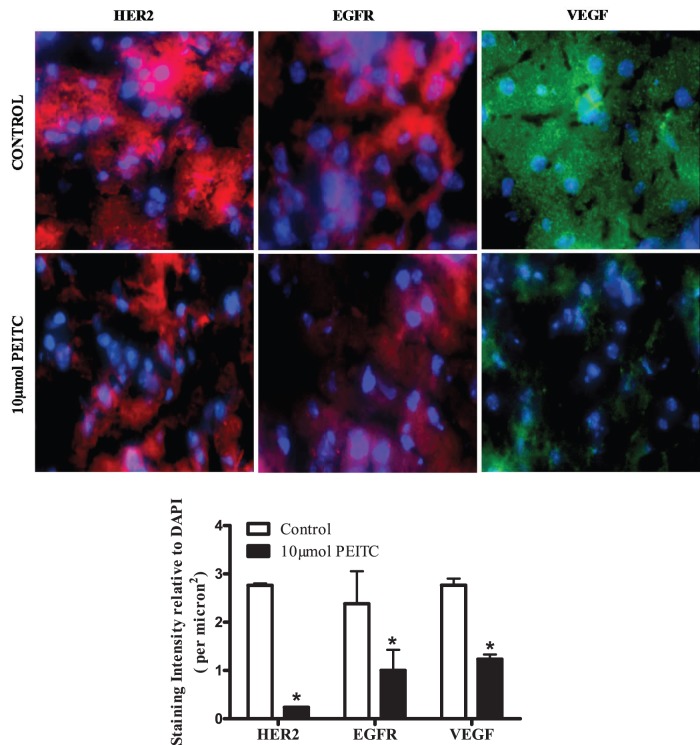
Effect of PEITC on the expression of HER2, EGFR and VEGF. The brain sections from control and PEITC treated groups were immunostained with HER2, EGFR, VEGF antibodies and DAPI for nuclear staining after fixation, permeabilization and blocking the tumor section. The images were taken using fluorescence microscope (Olympus Inc., Center valley, PA). The expression for HER2 (Red), EGFR (Red) and VEGF (Green) was quantitated using SlideBook software (Intelligent Imaging Innovations Inc., Denver, CO, USA). DAPI was used as internal control. * P<0.05, statistically different when compared with control. Results are presented as mean ± SD of triplicates.

After observing the reduced metastasis in PEITC treated mice, next step was to see whether PEITC could suppress the growth of metastasized tumors in brain. Two weeks after the breast tumor cells were lodged in the brain and started growing as indicated by the reappearance of luminescence in the brain, mice were treated with 10 µmol PEITC by oral gavage every day for 25 days (Fig. 2A). Our results reveal that the luminescence in the brain was relatively less in mice treated with PEITC, as compared to the control mice over the period of time (Fig. 2A). At the end of the experiment, tumor growth in the brain was suppressed by almost 50–55% by PEITC treatment (Fig. 2B), indicating the tumor growth suppressive effects of PEITC. Interestingly, luminescence was also observed at places other than the brain in the mice indicating that tumor cells metastasized to other sites. However, PEITC treatment substantially blocked the growth of these minor metastatic tumors as well (Fig. 2A). To evaluate the mechanism of the overall inhibitory effects of PEITC on the growth of metastatic breast tumors in the brain, brain sections from control and PEITC treated mice were examined by immunofluorescence. Our results show significantly reduced expressions of HER2 (by 90%), EGFR (by 50%) and VEGF (by 60%) in the brain sections of PEITC treated mice as compared to control mice (Fig. 3). Based on these observations, it is imperative that PEITC not only can block the metastasis but also can suppress the growth of metastasized breast tumors.

### Enhanced Survival of Mice Bearing Metastatic Breast Tumors by PEITC Treatment

**Figure 4:**
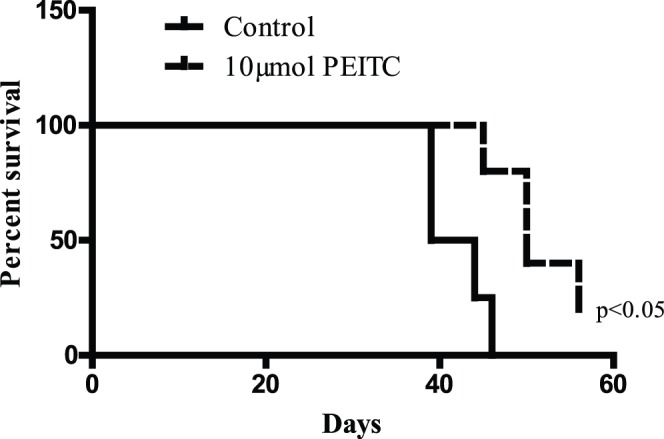
PEITC increased the survival of mice bearing tumors in brain. After two weeks of intra-cardiac injection of MDA-MB-231 (BR) cells, mice in treatment group were gavaged with 10 µmol PEITC orally every day till all the mice from control group were dead. Based on the data obtained, percent mice surviving at each time point were plotted using Kaplan Meier’s survival curve using Prism 5.0 (GraphPad software Inc., San Diego, CA). All the control mice bearing brain metastasis of MDA-MB-231 (BR) breast tumor cells died between 38–40 days after injection. * P<0.05, statistically different when compared with control.

Since PEITC significantly reduced the metastasis and growth of metastatic tumors, we hypothesized that PEITC could prolong the survival of breast tumor bearing mice. To test our hypothesis, we conducted a survival study in mice that were bearing metastatic breast tumors in the brain. Mice were injected with MDA-MB-231 (BR) cells through intracardiac route. Fourteen days after tumor cell injection, PEITC treatment started in the treatment group while the other group was given vehicle under similar conditions and served as control. Treatment continued until all the control mice died and survival curve was plotted using Kaplan Meier’s analysis. Our results show that mice in control group started dying from day 39 onwards (Fig. 4). The median survival time of mice in control group was 41.5 days (Fig. 4). However, the survival of PEITC-treated mice was prolonged by 20.5%, with a median survival time of 50 days. Interestingly, not all the mice died in PEITC-treated group by the end of the experiment. These observations suggest that due to its anti-metastatic potential, PEITC could be helpful in protracting the survival of breast cancer patients.

### Suppression of Cell Invasion by PEITC

**Figure 5:**
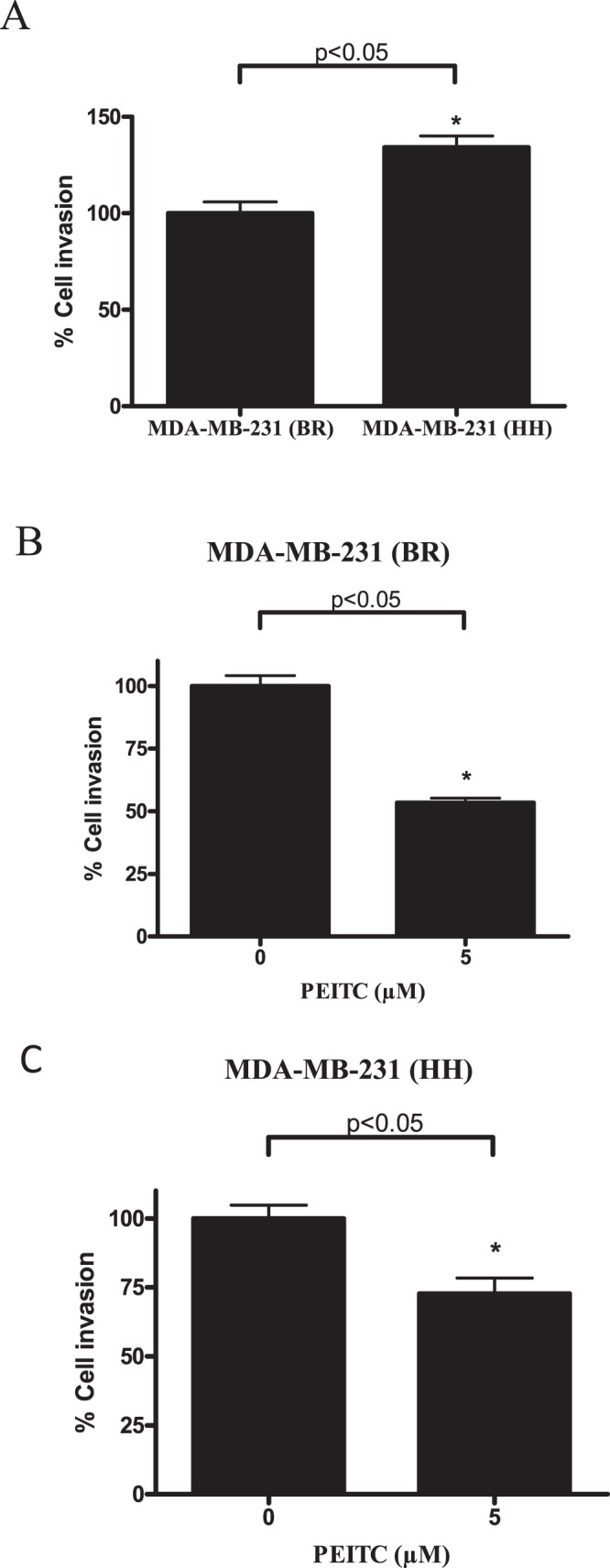
PEITC suppresses cell invasion. Invasion assay was performed using Boyden’s transwell chamber. (A) Comparison of the invasion potential of MDA-MB-231 (BR) (HH) cells with the MDA-MB-231 (BR) parent cells. Effect of 5 µM PEITC treatment on invasion of (B) MDA-MB-231 (BR) and (C) MDA-MB-231 (BR) high HER2 cells. Results are representative of three independent experiments. ^*^Statistically significant when compared with control (p<0.05).

Metastasis is a multistep process involving cell migration and invasion of tumor cells to distant organs from primary sites. The wound healing data shows inhibition of breast cancer cell migration by PEITC treatment (Fig. S1). The anti-cell invasive effects of PEITC were confirmed by cell invasion assay using Boyden’s chamber. In this experiment, effect of PEITC was evaluated on the capacity of breast cancer cells to invade through the membrane pores. Since HER2 has been known to be involved in tumor metastasis, we first wanted to see whether HER2 alone could increase the invasion of MDA-MB-231 (BR) cells. For this purpose, we used MDA-MB-231 (BR) cells stably overexpressing HER2 (MDA-MB-231 (HH)). We observed that HER2 overexpression increased the invasion of MDA-MB-231 (BR) cells by 1.4 fold (Fig. 5A). However, PEITC treatment suppressed the invasion of MDA-MB-231 (BR) cells by 50% (Fig. 5B) and MDA-MB-231 (HH) cells by 40% (Fig. 5C). In our recent studies we have demonstrated that PEITC suppresses the growth of MDA-MB-231 (HH) cells by reducing the expression of HER2 [32]. These results together with our previous observations suggest that PEITC targets HER2 to suppress the migration and invasion of breast cancer cells and hence metastasis.

## Discussion

Current study provides the first evidence for the anti-metastatic effects of PEITC *in vivo*. Observations from pretreatment model suggested that PEITC treatment significantly prevents the migration of breast cancer cell to brain *in vivo*. Based on these observations, we asked a question whether PEITC can suppress tumor growth in brain from the breast cancer cells that migrated to brain. Indeed oral administration of PEITC not only suppressed the growth of metastasized tumor in brain but also prolonged the survival of tumor bearing mice. The wound healing and Transwell cell invasion assay also suggested that PEITC suppresses cell migration and invasion of MDA-MB-231 (BR) breast cancer cells. Although, the MDA-MB-231 (BR) cells overexpressing HER2 have higher invasive potential, PEITC effectively suppressed migration of these cells as well. Our results indicate that PEITC can suppress metastasis of breast cancer cells in a preclinical model and provide further rationale for the clinical investigation of PEITC.

Breast cancer is a diverse disease, where almost 10–15% patients suffer from distant metastasis usually within three years of initial primary tumor [7], [8]. Brain is one of the preferential sites of breast cancer metastasis. Importantly, due to the inability of most therapeutics to cross blood brain barrier, the brain virtually becomes an incubation site for metastasized breast cancer cells. In most of the breast cancer patients, these tumor cells usually reside in brain un-diagnosed due to their undetectable size or dormancy. About 30% of patients revealed CNS metastasis of breast cancer after death [41]. In our current study, we observed that PEITC treatment reduced the number of MDA-MB-231 (BR) breast cancer cells reaching the brain by 50% in mice. Since PEITC also suppressed the proliferation of MDA-MB-231 (BR) cells (Fig. S2), cytotoxic effects of PEITC on these cells while in circulation cannot be ruled out. This outcome provides an indication that PEITC effectively reduces cancer cell migration to brain, hence suppresses tumor metastasis. There are reports suggesting few treatment options for preventing breast cancer metastasis to bones [42], [43], but this is the first study where the prevention of brain metastasis has been shown by a chemical inhibitor present in commonly used vegetables.

The brain tumor developed from metastasized cells can be treated by surgery or radiation therapy [44], [45]. However, these treatment options are available only after the diagnoses of brain tumors, whereas in many cases these metastasized tumors remain undiagnosed for long time. Considering the fact that breast tumors cells may remain dormant or grow as micro-tumors that are undetectable for long time, it is necessary to develop modalities to suppress the growth of metastasized tumors. The present study suggests the potential of PEITC in suppressing the growth of tumors in brain. We observed about 50–55% suppression of tumor burden in brains of mice treated with PEITC after intra-cardiac injection of MDA-MB-231 (BR) breast cancer cells. In addition, PEITC also suppressed the growth of few non-specific metastatic tumors in this model. This provides a direct evidence of the *in vivo* cytotoxic effects of PEITC on the growth of breast tumor cells at various metastatic sites in mice including brain.

Clinical testing for the presence of HER2 status has become a standard practice after the diagnosis of breast cancer. This can be explained by the increasing evidence for the role of HER2 in breast cancer progression and metastasis [10], [11], [46]. Palmieri et al. have shown a direct role of HER2 overexpression with that of increased metastatic outgrowth of breast cancer cells in brain [10]. Our recent studies demonstrated that PEITC can suppress the growth of breast tumor cells *in vitro* and *in vivo* by targeting HER2 [32]. In the present study, the immunofluorescence data showed significantly reduced staining of HER2 in the brain of mice treated with PEITC. It is possible that PEITC suppresses brain metastasis due to HER2 inhibition in mice brain. Our data provides a new direction for exploring the possible role of brain HER2 in providing a suitable microenvironment to breast cancer cells.

The role of vascular endothelial growth factor (VEGF) in breast cancer metastasis has been already established in several studies [13], [47]–[49]. We observed reduced expression of VEGF in the brains of mice treated with PEITC. These results suggest that PEITC elicits anti-metastatic effects possibly by suppressing VEGF levels, consistent with observation made earlier by other groups [50], [51]. Epidermal growth factor receptor has been long associated with metastasis [52]–[54]. Suppression of EGFR by 10 µM PEITC has been shown in prostate cancer cells by Kim et al [55]. In agreement with these studies, our studies also indicate EGFR suppression in the brains of mice treated with PEITC. Taken together, these observations suggest that suppression of breast cancer metastasis to brain by PEITC could be associated with inhibition of HER2, EGFR and VEGF. However, detailed studies are required to mechanistically establish this correlation.

The prognosis of breast cancer patients with brain metastasis is generally poor and the survival of the patients ranges from 3–5 months [56]–[58]. Our data shows that PEITC enhanced the median survival of mice by almost 21% that had metastasized breast tumors.

PEITC has been reported to have high oral bioavailability. The oral bioavailability of PEITC in rats was found to be 115 and 93% with doses of 10 µmol/kg and 100 µmol/kg respectively [59]. Based on these studies, it appears that the concentrations of PEITC used in our experiments can be achieved by oral administration. Conaway et al. achieved the concentration of about 2.07pmol/mg of PEITC in the brain tissue by gavaging 10 µmol/kg PEITC in each mouse [30]. This observation indicated that PEITC can reach brain in substantial amounts indicating its availability in brain tissue. It is conceivable that the growth suppression of metastatic tumors in the brain of mice treated with PEITC could be due to the fact that PEITC can reach brain through systemic circulation, cross blood brain barrier and elicit anti-tumor effects.

### Conclusions

We recently demonstrated the anti-proliferative effects of PEITC in breast cancer cells and that the effect of PEITC was more pronounced in HER2 positive breast cancer cells *in vitro* and *in vivo* [32]. Our current study presents a novel role of PEITC in preventing and suppressing breast cancer metastasis *in vivo* possibly by suppressing HER2, EGFR and VEGF, which are known to promote cell motility. Taken together, the results from our study indicate that PEITC suppresses brain metastasis of breast cancer cells.

## Supporting Information

Figure S1 (EPS)

Figure S2 (EPS)

## References

[1] Mehlen P, Puisieux A (2006) Metastasis: a question of life or death. Nat Rev Cancer 6: 449–458. 16723991 10.1038/nrc1886

[2] O'Shaughnessy J (2005) Extending survival with chemotherapy in metastatic breast cancer. Oncologist 10 Suppl 320–29. 16368868 10.1634/theoncologist.10-90003-20

[3] Jemal A, Bray F (2011) Center MM, Ferlay J, Ward E, et al (2011) Global cancer statistics. CA Cancer J Clin 61: 69–90. 21296855 10.3322/caac.20107

[4] Chang EL, Lo S (2003) Diagnosis and management of central nervous system metastases from breast cancer. Oncologist 8: 398–410. 14530493 10.1634/theoncologist.8-5-398

[5] Lassman AB, DeAngelis LM (2003) Brain metastases. Neurol Clin 21: 1–23, vii. 10.1016/s0733-8619(02)00035-x 12690643

[6] Frisk G, Svensson T, Backlund LM, Lidbrink E, Blomqvist P, et al (2012) Incidence and time trends of brain metastases admissions among breast cancer patients in Sweden. Br J Cancer 106: 1850–1853. 22531629 10.1038/bjc.2012.163 PMC3364124

[7] Lin NU, Bellon JR, Winer EP (2004) CNS metastases in breast cancer. J Clin Oncol 22: 3608–3617. 15337811 10.1200/JCO.2004.01.175

[8] Weigelt B, Peterse JL, van 't Veer LJ (2005) Breast cancer metastasis: markers and models. Nat Rev Cancer 5: 591–602. 16056258 10.1038/nrc1670

[9] Fidler IJ, Balasubramanian K, Lin Q, Kim SW, Kim SJ (2010) The brain microenvironment and cancer metastasis. Mol Cells 30: 93–98. 20799011 10.1007/s10059-010-0133-9 PMC11812924

[10] Palmieri D, Bronder JL, Herring JM, Yoneda T, Weil RJ, et al (2007) Her-2 overexpression increases the metastatic outgrowth of breast cancer cells in the brain. Cancer Res 67: 4190–4198. 17483330 10.1158/0008-5472.CAN-06-3316

[11] Dieras V, Pierga JY (2011) Brain metastasis of breast tumors and blood brain barrier. Bull Cancer 98: 385–389. 21527366 10.1684/bdc.2011.1336

[12] Pazaiti A, Fentiman IS (2011) Basal phenotype breast cancer: implications for treatment and prognosis. Womens Health (Lond Engl) 7: 181–202. 21410345 10.2217/whe.11.5

[13] Fidler IJ (2011) The role of the organ microenvironment in brain metastasis. Semin Cancer Biol 21: 107–112. 21167939 10.1016/j.semcancer.2010.12.009

[14] Brown JM, Giaccia AJ (1998) The unique physiology of solid tumors: opportunities (and problems) for cancer therapy. Cancer Res 58: 1408–1416. 9537241

[15] Stewart PA, Hayakawa K, Farrell CL, Del Maestro RF (1987) Quantitative study of microvessel ultrastructure in human peritumoral brain tissue. Evidence for a blood-brain barrier defect. J Neurosurg 67: 697–705. 3668638 10.3171/jns.1987.67.5.0697

[16] Yano S, Shinohara H, Herbst RS, Kuniyasu H, Bucana CD, et al (2000) Expression of vascular endothelial growth factor is necessary but not sufficient for production and growth of brain metastasis. Cancer Res 60: 4959–4967. 10987313

[17] Palmer S (1985) Diet, nutrition, and cancer. Prog Food Nutr Sci 9: 283–341. 3010379

[18] Boggs DA, Palmer JR, Wise LA, Spiegelman D, Stampfer MJ, et al (2010) Fruit and vegetable intake in relation to risk of breast cancer in the Black Women's Health Study. Am J Epidemiol 172: 1268–1279. 20937636 10.1093/aje/kwq293 PMC3025632

[19] Lee SA, Fowke JH, Lu W, Ye C, Zheng Y, et al (2008) Cruciferous vegetables, the GSTP1 Ile105Val genetic polymorphism, and breast cancer risk. Am J Clin Nutr 87: 753–760. 18326615 10.1093/ajcn/87.3.753

[20] Butler LM, Wu AH, Wang R, Koh WP, Yuan JM, et al (2010) A vegetable-fruit-soy dietary pattern protects against breast cancer among postmenopausal Singapore Chinese women. Am J Clin Nutr 91: 1013–1019. 20181808 10.3945/ajcn.2009.28572 PMC2844682

[21] Daxenbichler ME, VanEtten CH (1977) Glucosinolates and derived products in cruciferous vegetables: gas-liquid chromatographic determination of the aglucon derivatives from cabbage. J Assoc Off Anal Chem 60: 950–953. 893314

[22] Brown KK, Hampton MB (2011) Biological targets of isothiocyanates. Biochim Biophys Acta 1810: 888–894. 21704127 10.1016/j.bbagen.2011.06.004

[23] Hecht SS (2000) Inhibition of carcinogenesis by isothiocyanates. Drug Metab Rev 32: 395–411. 11139137 10.1081/dmr-100102342

[24] Wu XJ, Hua X (2007) Targeting ROS: selective killing of cancer cells by a cruciferous vegetable derived pro-oxidant compound. Cancer Biol Ther 6: 646–647. 17387274 10.4161/cbt.6.5.4092

[25] Cheung KL, Kong AN (2010) Molecular targets of dietary phenethyl isothiocyanate and sulforaphane for cancer chemoprevention. AAPS J 12: 87–97. 20013083 10.1208/s12248-009-9162-8 PMC2811646

[26] Jakubikova J, Cervi D, Ooi M, Kim K, Nahar S, et al (2011) Anti-tumor activity and signaling events triggered by the isothiocyanates, sulforaphane and phenethyl isothiocyanate, in multiple myeloma. Haematologica 96: 1170–1179. 21712538 10.3324/haematol.2010.029363 PMC3148911

[27] Gao N, Budhraja A, Cheng S, Liu EH, Chen J, et al (2011) Phenethyl isothiocyanate exhibits antileukemic activity in vitro and in vivo by inactivation of Akt and activation of JNK pathways. Cell Death Dis 2: e140. 21472003 10.1038/cddis.2011.22 PMC3122055

[28] Wu CL, Huang AC, Yang JS, Liao CL, Lu HF, et al (2011) Benzyl isothiocyanate (BITC) and phenethyl isothiocyanate (PEITC)-mediated generation of reactive oxygen species causes cell cycle arrest and induces apoptosis via activation of caspase-3, mitochondria dysfunction and nitric oxide (NO) in human osteogenic sarcoma U-2 OS cells. J Orthop Res 29: 1199–1209. 21374707 10.1002/jor.21350

[29] Yang MD, Lai KC, Lai TY, Hsu SC, Kuo CL, et al (2010) Phenethyl isothiocyanate inhibits migration and invasion of human gastric cancer AGS cells through suppressing MAPK and NF-kappaB signal pathways. Anticancer Res 30: 2135–2143. 20651362

[30] Conaway CC, Jiao D, Kohri T, Liebes L, Chung FL (1999) Disposition and pharmacokinetics of phenethyl isothiocyanate and 6-phenylhexyl isothiocyanate in F344 rats. Drug Metab Dispos 27: 13–20. 9884304

[31] Wu X, Zhu Y, Yan H, Liu B, Li Y, et al (2010) Isothiocyanates induce oxidative stress and suppress the metastasis potential of human non-small cell lung cancer cells. BMC Cancer 10: 269. 20534110 10.1186/1471-2407-10-269 PMC2891640

[32] Gupta P, Srivastava SK (2012) Anti-tumor activity of phenethyl isothiocyanate in HER2 positive breast cancer models. BMC Med 10: 80. 22824293 10.1186/1741-7015-10-80 PMC3412708

[33] Kandala PK, Srivastava SK (2012) Diindolylmethane mediated Gli1 Suppression Induces Anoikis in Ovarian Cancer cells in vitro and blocks tumor formation ability in vivo. J Biol Chem. 10.1074/jbc.M112.351379 PMC3436512 22773833

[34] Kandala PK, Srivastava SK (2012) Regulation of macroautophagy in ovarian cancer cells in vitro and in vivo by controlling glucose regulatory protein 78 and AMPK. Oncotarget 3: 435–449. 22564965 10.18632/oncotarget.483 PMC3380578

[35] Yoneda T, Williams PJ, Hiraga T, Niewolna M, Nishimura R (2001) A bone-seeking clone exhibits different biological properties from the MDA-MB-231 parental human breast cancer cells and a brain-seeking clone in vivo and in vitro. J Bone Miner Res 16: 1486–1495. 11499871 10.1359/jbmr.2001.16.8.1486

[36] Palmieri D, Lockman PR, Thomas FC, Hua E, Herring J, et al (2009) Vorinostat inhibits brain metastatic colonization in a model of triple-negative breast cancer and induces DNA double-strand breaks. Clin Cancer Res 15: 6148–6157. 19789319 10.1158/1078-0432.CCR-09-1039 PMC7356672

[37] Lockman PR, Mittapalli RK, Taskar KS, Rudraraju V, Gril B, et al (2010) Heterogeneous blood-tumor barrier permeability determines drug efficacy in experimental brain metastases of breast cancer. Clin Cancer Res 16: 5664–5678. 20829328 10.1158/1078-0432.CCR-10-1564 PMC2999649

[38] Conley FK (1979) Development of a metastatic brain tumor model in mice. Cancer Res 39: 1001–1007. 427739

[39] Sahu RP, Batra S, Srivastava SK (2009) Activation of ATM/Chk1 by curcumin causes cell cycle arrest and apoptosis in human pancreatic cancer cells. Br J Cancer 100: 1425–1433. 19401701 10.1038/sj.bjc.6605039 PMC2694438

[40] Boreddy SR, Sahu RP, Srivastava SK (2011) Benzyl isothiocyanate suppresses pancreatic tumor angiogenesis and invasion by inhibiting HIF-alpha/VEGF/Rho-GTPases: pivotal role of STAT-3. PLoS One 6: e25799. 22016776 10.1371/journal.pone.0025799 PMC3189946

[41] Tsukada Y, Fouad A, Pickren JW, Lane WW (1983) Central nervous system metastasis from breast carcinoma. Autopsy study. Cancer 52: 2349–2354. 6640506 10.1002/1097-0142(19831215)52:12<2349::aid-cncr2820521231>3.0.co;2-b

[42] Coleman RE (2012) Bone cancer in 2011: Prevention and treatment of bone metastases. Nat Rev Clin Oncol 9: 76–78. 10.1038/nrclinonc.2011.198 22182971

[43] Jeong J, Lee KS, Choi YK, Oh YJ, Lee HD (2011) Preventive effects of zoledronic acid on bone metastasis in mice injected with human breast cancer cells. J Korean Med Sci 26: 1569–1575. 22147993 10.3346/jkms.2011.26.12.1569 PMC3230016

[44] Tsao MN, Lloyd N, Wong RK, Chow E, Rakovitch E, et al (2012) Whole brain radiotherapy for the treatment of newly diagnosed multiple brain metastases. Cochrane Database Syst Rev 4: CD003869. 10.1002/14651858.CD003869.pub3 PMC6457607 22513917

[45] Padovani L, Muracciole X, Regis J (2012) gamma knife radiosurgery of brain metastasis from breast cancer. Prog Neurol Surg 25: 156–162. 22236677 10.1159/000331189

[46] Liu XL, Peng CW, Chen C, Yang XQ, Hu MB, et al (2011) Quantum dots-based double-color imaging of HER2 positive breast cancer invasion. Biochem Biophys Res Commun 409: 577–582. 21609713 10.1016/j.bbrc.2011.05.052

[47] Claffey KP, Robinson GS (1996) Regulation of VEGF/VPF expression in tumor cells: consequences for tumor growth and metastasis. Cancer Metastasis Rev 15: 165–176. 8842488 10.1007/BF00437469

[48] Li X, Dang X, Sun X (2012) Expression of survivin and VEGF-C in breast cancer tissue and its relation to lymphatic metastasis. Eur J Gynaecol Oncol 33: 178–182. 22611959

[49] De Luca A, Lamura L, Gallo M, Maffia V, Normanno N (2012) Mesenchymal stem cells-derived interleukin-6 and vascular endothelial growth factor promote breast cancer cell migration. J Cell Biochem 113: 3363–3370. 22644871 10.1002/jcb.24212

[50] Xu C, Shen G, Chen C, Gelinas C, Kong AN (2005) Suppression of NF-kappaB and NF-kappaB-regulated gene expression by sulforaphane and PEITC through IkappaBalpha, IKK pathway in human prostate cancer PC-3 cells. Oncogene 24: 4486–4495. 15856023 10.1038/sj.onc.1208656

[51] Wang XH, Cavell BE, Syed Alwi SS, Packham G (2009) Inhibition of hypoxia inducible factor by phenethyl isothiocyanate. Biochem Pharmacol 78: 261–272. 19376091 10.1016/j.bcp.2009.04.010

[52] Lau SK, Shields DJ, Murphy EA, Desgrosellier JS, Anand S, et al (2012) EGFR-Mediated Carcinoma Cell Metastasis Mediated by Integrin alphavbeta5 Depends on Activation of c-Src and Cleavage of MUC1. PLoS One 7: e36753. 22586492 10.1371/journal.pone.0036753 PMC3346745

[53] Nie F, Yang J, Wen S, An YL, Ding J, et al (2012) Involvement of epidermal growth factor receptor overexpression in the promotion of breast cancer brain metastasis. Cancer 118: 5198–5209. 22510844 10.1002/cncr.27553

[54] Nickerson NK, Mohammad KS, Gilmore JL, Crismore E, Bruzzaniti A, et al (2012) Decreased Autocrine EGFR Signaling in Metastatic Breast Cancer Cells Inhibits Tumor Growth in Bone and Mammary Fat Pad. PLoS One 7: e30255. 22276166 10.1371/journal.pone.0030255 PMC3261896

[55] Kim JH, Xu C, Keum YS, Reddy B, Conney A, et al (2006) Inhibition of EGFR signaling in human prostate cancer PC-3 cells by combination treatment with beta-phenylethyl isothiocyanate and curcumin. Carcinogenesis 27: 475–482. 16299382 10.1093/carcin/bgi272

[56] Fokstuen T, Wilking N, Rutqvist LE, Wolke J, Liedberg A, et al (2000) Radiation therapy in the management of brain metastases from breast cancer. Breast Cancer Res Treat 62: 211–216. 11072785 10.1023/a:1006486423827

[57] Boogerd W, Vos VW, Hart AA, Baris G (1993) Brain metastases in breast cancer; natural history, prognostic factors and outcome. J Neurooncol 15: 165–174. 8509821 10.1007/BF01053937

[58] Patanaphan V, Salazar OM, Risco R (1988) Breast cancer: metastatic patterns and their prognosis. South Med J 81: 1109–1112. 3420442

[59] Ji Y, Kuo Y, Morris ME (2005) Pharmacokinetics of dietary phenethyl isothiocyanate in rats. Pharm Res 22: 1658–1666. 16180123 10.1007/s11095-005-7097-z

